# How School Contexts Shape the Relations Among Adolescents’ Beliefs, Peer Victimization, and Depressive Symptoms

**DOI:** 10.1111/jora.12558

**Published:** 2020-05-09

**Authors:** Tessa M. L. Kaufman, Hae Yeon Lee, Aprile D. Benner, David S. Yeager

**Affiliations:** ^1^ University of Groningen; ^2^ University of Texas at Austin

## Abstract

The present research examined how school contexts shape the extent to which beliefs about the potential for change (implicit theories) interact with social adversity to predict depressive symptoms. A preregistered multilevel regression analysis using data from 6,237 ninth‐grade adolescents in 25 U.S. high schools showed a three‐way interaction: Implicit theories moderated the associations between victimization and depressive symptoms only in schools with high levels of school‐level victimization, but not in schools with low victimization levels. In high‐victimization schools, adolescents who believed that people cannot change (an entity theory of personality) were more depressed when they were victimized more frequently. Thus, the mental health correlates of adolescents’ implicit theories depend on both personal experiences and the norms in the context.

Peer victimization in adolescence can contribute to a vicious cycle whereby victimized adolescents become distressed and suffer poorer mental health, which in turn can invite further victimization occurrences from peers over time (Kaufman, Kretschmer, Huitsing, & Veenstra, 2020; Reijntjes, Kamphuis, Prinzie, & Telch, 2010; Sentse, Prinzie, & Salmivalli, 2017). A high scientific and societal priority has therefore been to identify potentially modifiable processes that contribute to victimization‐related mental health problems. Two such processes include adolescents’ *implicit theories* and the norms in the *school context*. Both of these can shape the meaning that adolescents make out of their peer victimization experiences, and these may interact when predicting outcomes.

Adolescents’ *implicit theories* about the malleability versus fixedness of human social or moral characteristics are a social–cognitive factor that shows promise for understanding the link between victimization and mental health. Implicit theories guide adolescents’ interpretations of, and coping responses to, events in their social worlds, especially socially stressful, evaluative events, such as peer rejection, exclusion, and relational aggression (Dweck & Leggett, 1988; Molden & Dweck, 2006; Yeager, Miu, Powers, & Dweck, 2013). There is considerable empirical evidence that implicit theories can affect various psychological processes such as acute stress responses (Yeager, Lee, & Jamieson, 2016), and implicit theories can strengthen associations between victimization and depressive symptoms (Rudolph, 2010; Yeager et al., 2014; Yeager, Trzesniewski, & Dweck, 2013). Adolescents who hold more of an *entity theory of personality*—the belief that people’s socially relevant traits cannot change—are more likely to see victimization as carried out by and to people who have little potential for change. This fixed‐trait attribution can lead adolescents to worry about peer victimization enduring perpetually. In contrast, adolescents who hold more of an *incremental theory of personality* believe that people have the capacity to change and thus may think that victimization is done by and to people who can change and improve over time; these individuals tend to see their future outlook as more hopeful. In prior research, youth taught an incremental theory of personality was less distressed following social exclusion (Yeager et al., 2014) and reported lower depressive symptoms following victimization (Yeager, Trzesniewski, et al., 2013).

Despite the evidence that implicit theories can improve mental health and buffer against the emotional sequelae of victimization encounters under some conditions, far too little is known about how, why, and under what conditions that link might appear. Notably, to date studies have not incorporated the role of social context that may afford different construals of victimization events (Walton & Yeager, 2020). Further, existing studies have rarely separated the roles of *within*‐context variability in victimization (how victimized I am relative to peers in my school) versus *between*‐context victimization (how often people in my school are victimized in general, relative to other schools). This distinction is relevant because implicit theories might have greater impacts in some social contexts than in others (Yeager et al., 2019). Particularly, implicit theories become more relevant in situations in which individuals experience greater social stress (Yeager et al., 2014) or undergo an “ego threat,” which is defined as an event or communication that has unfavorable implications about the self regard, such as one’s abilities, traits, or social status (Burnette, O’Boyle, VanEpps, Pollack, & Finkel, 2012).

Whether peer victimization is indeed perceived as a social threat likely depends on the average level of victimization in victims’ social contexts, thus between‐context victimization. Intuitively, if you are the only person being victimized in your school it could mean something different, in terms of your present or future relationships with others and your self‐esteem, than being in a school in which most students are victimized at one time or another (Garandeau, Lee, & Salmivalli, 2018; Huitsing, Veenstra, Sainio, & Salmivalli, 2012; Schacter & Juvonen, 2016, 2018a). Consequently, the extent to which implicit theories shape the meaning and the mental health effects of victimization may be heterogeneous across social contexts that differ in the extent to which victimization is common. In the present research, we test this directly.

## Implicit Theories × Context Interactions On Mental Health Correlates of Victimization

A focus on contextual heterogeneity of the role of implicit theories calls for an integration of psychological and sociological perspectives (Yeager et al., 2019). The psychological perspective describes how implicit theories shape the meaning of victimization experiences, whereas the sociological perspective considers how informal social contexts, such as exposure to school‐level victimization, strengthen or weaken such individual processes.

As noted, implicit theories are a social–cognitive frame that individuals use to make sense of and cope with adverse events. Similar to other social–cognitive processes—such as hostile attribution schemas (Dodge, 2006) or negative inferential styles (Giollabhui et al., 2018)—implicit theories shape predictable tendencies in individuals’ causal inferences about the person (e.g., “they are mean people”; “I am a loser”) and the situation (e.g., “this bullying will never stop”). Therefore, implicit theories influence individuals’ coping responses to the adverse event, resulting in hopelessness or depressive symptoms (Yeager et al., 2014). However, implicit theories are different from hostile schemas and negative inferential styles because they do not always arise from negative experiences and do not necessarily include overall negative views about the social world. Instead, implicit theories shape the meaning of peer provocations of ambiguous intent but clearly negative consequences. At these moments, implicit theories create a different, subjective psychological reality through which adolescents judge their own or others’ global character, status, or future behavior from minor cues.

This role of implicit theories in affecting meaning‐making processes out of individual experiences such as victimization may depend on the social context in which victimization occurs. Some recent research has shed lights on the contextual heterogeneity of implicit theories in leading to divergent developmental outcomes in the domain of academic performance. For example, in school contexts where peer norms are aligned with an implicit theory taught in an intervention, the intervention had positive effects, but there were no effects when the peer norms were inconsistent with the implicit theories (Yeager et al., 2019). Contexts make possible, or they foreclose, particular ways of experiencing, interpreting, and responding to events (Walton & Yeager, 2020). Thus, implicit theories become more or less relevant depending on whether they are afforded by the social context.

Here, we build on this past research and focus on a potential interaction between individuals’ beliefs and their school contexts. There are two contrasting hypotheses about the direction of the effect of school‐average victimization on depressive symptoms. On the one hand, the effects of implicit theories may be stronger in contexts where victimization is *less* common (*few fellow victims* hypothesis), because people may harshly judge themselves (and feel more depressed) when they are the only ones getting victimized. On the other hand, implicit theories may be more relevant to victims in schools in which victimization occurs *more* frequently (*many fellow victims* hypothesis), because the double adversity triggers the entity theory more strongly. Because the existing literature provides support for processes in either of the directions, it is important to conduct a confirmatory study to distinguish among them—something we do here.

### Few Fellow Victims Hypothesis

The first, “few fellow victims” hypothesis holds that implicit theories might primarily affect victimized adolescents’ depressive symptoms in schools where victimization is less common (i.e., with few fellow victims). Implicit theories play a greater role in adolescents’ adjustment in contexts where they perceive an ego threat, such as those that emphasize personal failure (Burnette et al., 2012), and victims may particularly experience feelings of failure when in contexts with few fellow victims (Garandeau et al., 2018; Huitsing et al., 2019; Juvonen, Schacter, Sainio, & Salmivalli, 2016). The reason why comes from social comparison theory (Festinger, 1954), which suggests that people seek to evaluate themselves by comparing their stressful experiences to those of relevant others. For adolescents, those relevant others are generally their peers. When adolescents are worse off than their peers, they tend to attribute the cause of their experiences to their own self‐deficiencies (Weiner, 1985). Thus, in a situation in which adolescents are targeted for victimization events but their peers are not, social comparisons could result in internal attributions for victimization, resulting in poorer mental health outcomes.

More recently, bullying researchers have called this phenomenon “the healthy context paradox,” which refers to the maladaptive effects of small groups of individuals who are worse off in an otherwise positive environment (Huitsing et al., 2019; Salmivalli, 2018). Empirical work on context‐level moderators of the impact of victimization supports this perspective. When only few others are victimized (Schacter & Juvonen, 2016) or when victims’ close friends are less bullied (Schacter & Juvonen, 2018a), victims tended to blame themselves more. Moreover, persistent victims felt more distressed in classrooms where the proportion of victims had decreased (Garandeau et al., 2018) or in schools with an effective antibullying intervention (Huitsing et al., 2019). Overall, being one of the only victims can result in greater internal causal attributions or negative feelings about the self.

These psychological processes can be considered as feelings of failure and thus characterize the “ego threat” that can strengthen the associations between implicit theories and negative mental health outcomes. According to this *few fellow victims* hypothesis, adolescents who endorse an entity theory will perceive their experiences of failure as being fixed, and thus become more hopeless and depressed (Abramson & Metalsky, 1989), believing that victimization will be perpetual. Adolescents who endorse an incremental theory of personality may not necessarily grow hopeless, but may view possible internal causes as factors that can be changed to improve their situation and persist in trying to do so (Burnette et al., 2012; Dweck & Leggett, 1988). Adolescents with an incremental theory try to actively improve their future outcomes through making targeted efforts, learning, and striving to strengthen their abilities or attitudes when faced with setbacks such as victimization (Molden & Dweck, 2006). Thus, victims with an incremental theory in contexts with few fellow victims may view these perceived internal causes as changeable, making then more hopeful. To summarize, the few fellow victims hypothesis posits that implicit theories may predict the sequelae of victimization, such as depressive symptoms, mainly in contexts with few fellow victims, because these contexts make people personalize their social failures more strongly.

### Many Fellow Victims Hypothesis

Alternatively, implicit theories could be of most relevance in contexts where victimized adolescents are surrounded by many fellow victims. This literature focuses on how environments shape hostile perceptions about others, instead of about the self, resulting in hopelessness and thus depressive symptoms. From this perspective, being victimized in an unsafe, high‐victimization environment is harmful because it creates the perception that escaping victimization is almost inevitable and that one needs to be constantly vigilant to a strong threat. It was shown that witnessing bullying at school predicted risks to mental health over and above direct experiences of victimization (Rivers, Poteat, Noret, & Ashurst, 2009), that schools in which aggression was considered more normative were linked to greater individual aggression (Henry et al., 2000), and that victims in schools with lower prosocial (i.e., helping each other) norms felt more distressed and perceived their school climate as more unsafe (Schacter & Juvonen, 2018b).

This environmental distress from chronic exposure to bullying may be experienced differentially depending on individuals’ implicit theories. An entity theory generally makes individuals more inclined to perceive others’ behaviors and intentions as hostile (Yeager, Miu, et al., 2013) and is characterized by the belief that people like bullies will not change. Therefore, adolescents who endorse an entity theory will consider their own experiences of being victimized and indirect observations of frequent bully attacks in the school as “social proof” of their beliefs, and exacerbate the negative consequences of an entity theory. Again, this would be in line with research showing that implicit theories had most impact when peer norms aligned with them, and was thus congruent with the belief system (Yeager et al., 2019).

### The Current Study

We extend previous theory and research on the relations among implicit theories, peer victimization, and depressive symptoms (Yeager, Trzesniewski, et al., 2013) by examining how this interaction depends on school contexts with data from a large sample of ninth‐grade adolescents attending 25 U.S. public high schools (*N* = 6,237). The context factor we focused on was the average levels of victimization in the school.

Our approach was novel in two ways. First, it moved the field on implicit theories forward by illustrating the role of school context (also see Yeager et al., 2019), but in the mental health domain. Second, our approach shows how the link between peer victimization and mental health can depend both on personal beliefs and on norms in the context, and how this can be studied, which sets the stage for more multifaceted intervention research.

We hypothesized that adolescents’ higher individual‐level victimization and entity theories (versus incremental theories) would predict greater depressive symptoms (H1). Further, we expected that holding an entity theory (versus an incremental theory) would predict a stronger association between victimization and depressive symptoms (H2). Last, and most importantly, we hypothesized that this overall pattern would depend on the prevalence of victimization in adolescents’ school context (H3). For H3, our primary hypothesis, we allowed the data to distinguish between two possibilities that, prior to seeing the results, seemed equally plausible: that implicit theories could play a larger role in schools where victimization is less common, or that implicit theories could play a larger role in schools where victimization is more common. In order to constrain researcher degrees of freedom and reduce the chance of false‐positive results, we preregistered all data processing decisions, hypotheses, and statistical models prior to accessing the dataset (https://osf.io/4qb5d).

## Method

### Participants

Data stem from the Texas Longitudinal Study of Adolescent Stress Resilience: Saturated Schools Sample. This sample included a total of *N* = 6,237 ninth‐grade adolescents (49% boys; within‐school range 31–61% boys) who were recruited from 25 high schools located in 16 states across the United States. The schools were subsampled from an existing national probability sample of schools, the National Study of Learning Mindsets (or NSLM, Yeager, 2019), with the 2017–2018 cohort of 9th graders, which was two cohorts after the initial cohort of 2015–2016 ninth‐grade students who participated in the NSLM. One independent company (ICF) drew the sample, recruited schools, arranged for treatment delivery, supervised and implemented the data collection protocol, obtained administrative data, and cleaned and merged data. This study used a convenience subset of schools that had specific characteristics and had previously participated in the NSLM. From the initial pool of 76 schools, candidates were ranked by the following characteristics: ease to work with, perceived interest to continue the study, ninth‐grade enrollment over 125, and their election not to use active parental consent in the NSLM. A list of 42 schools emerged for potential participation, and each school was approached about participation until 25 schools agreed to participate. On average, almost all students (95.4%, on average) from each school participated in the study (*M* = 339 students per school, ranging from 141 to 594).

Students participated in this study as a part of a broader program evaluation, and so passive consent procedures were used with students’ parents. Parents were notified in a letter about the study and its aims, and had the opportunity to withdraw their students. Active assent was obtained from all students during the survey. Any student who declined to participate was removed from the dataset and was not recruited subsequently to participate.

The present sample of schools is not strictly representative, but it is highly heterogeneous and inclusive of the diversity of school contexts in the United States and therefore the sample is informative for testing the present study’s hypotheses about the role of context. Schools were diverse with regard to students’ self‐reported ethnicity/race demographics (for estimates per school, see Table A3). Almost half (49.5%) of the adolescents identified as White/European American, 13.2% as Hispanic/Latinx, 11.9% as Black/African American, 5.6% as Asian/Asian American, 5.8% as Middle Eastern, 3.6% as Native American Indian, 1.7% as Hawaiian/Pacific Islanders, and 8.6% as another race/ethnicity.

In terms of maternal education as an indicator of family socioeconomic status, within schools, 10.3% said their mother did not finish high school, 15.7% said their mother finished high school without a college degree, 11.1% reported some college‐level courses, 7.5% reported a two‐year associate degree, 18.1% reported a 4‐year college degree, 10.0% reported a master’s degree, 3.3% said their mother completed a PhD or other professional degree, and 24.0% did not know their mother’s highest obtained degree (for estimates per school, see Table A4).

### Measures

#### Individual and School‐Average Victimization

These measures were assessed with six items about physical, verbal, and relational victimization experiences by other students in the last two weeks (1 = *never* to 5 = *a few times a week*; Prinstein, Boergers, & Vernberg, 2001). A sample item was as follows: “Another student threatened to hurt or beat me up.”

Validity analyses (Appendix 1) of the operationalization of the victimization measure on the school level supported the use of the aggregated mean score of all items of victimization. We performed validity analyses because aggregates of self‐reported victimization are sensitive to bias by students’ frame of reference (reference bias; Duckworth & Yeager, 2015). The aggregated school‐level mean score was associated with higher school suspensions (*r* = .34) and lower school quality ratings (according to greatschools.org; *r* = −.36), and slightly more discipline incidents (*r =* .14), which are considered more objective indicators of social safety and thus victimization levels. We assessed school‐average victimization with the aggregated mean scores across all six items (*M* = 1.34, *SD* = 0.01; range 1.22–1.47) and consistently used the mean to construct the individual measure of victimization (α = .79).

#### Depressive Symptoms

Depressive symptoms, the main outcome in this study, were assessed using the Children’s Depression Inventory (CDI, mean of 26 items, suicidal ideation item removed; Kovacs, 1992; α = .90). Each item asked participants to report which of three levels of a symptom described their feelings best in the past two weeks (e.g., 0 = “*I am sad once in a while,*” 1 = “*I am sad many times,*” and 2 = “*I am sad all the time*”). We analyzed the unweighted average of the items. The intraclass correlation was .02. Thus, most of the variance in depressive symptoms existed between individual students instead of schools. The design effect (*D*_eff_) that considers cluster size was D_eff_ = 5.98, which supports our planned multilevel approach (Muthén & Satorra, 1995).

#### Entity Theory of Personality

We assessed adolescents’ implicit beliefs about the malleability of status‐relevant social traits using an eight‐item scale previously validated with adolescent samples. Items overall measured the extent to which adolescents endorsed an entity theory of personality—the belief that status‐relevant social traits (e.g., bullies, victims, winners, and losers) are fixed and cannot change—or an incremental theory of personality—the belief that people’s status‐relevant traits can change. Four items focused on the malleability of bullies/victims and mean people (“jerks”) (Miu & Yeager, 2015; Yeager, Trzesniewski, et al., 2013), while the other four items focused on the malleability of social status more broadly, such as popularity (Lee & Yeager, 2019). Sample items include “*Bullies and victims are types of people that really can’t be changed*” and “*Popular people and unpopular people are types of people that really can’t be changed*” (6‐point scale, 1 = *Strongly disagree* to 6 = *Strongly agree*; α = .82). Higher composite scores reflect more of an entity theory, whereas lower scores reflect more of an incremental theory. The four items on personality were correlated strongly with the four items on social status (*r* = .55). Moreover, in line with previous research (Lee & Yeager, 2019), factor analysis showed support for a single latent factor with satisfactory loadings of all items (factor loadings .45–.74), χ^2^(17) = 435.22, *p* < .001, *RMSEA* = .06, 90% *CI* [.058, .068], *CFI* = .97, *TLI* = .96. Therefore, our primary analyses used a single composite score averaging all eight items.

#### Covariates

Adolescents’ self‐identified *gender* was assessed with a binary variable (0 = *male*, 1 = *female*) and entered as an individual‐level covariate. *School‐level achievement* was a latent variable used as a school‐level covariate, created from unweighted *z*‐scored average of state standardized test scores, PSAT scores, rates of taking and passing A.P. courses, and related achievement indicators (Tipton et al., 2019).

### Analytical Strategy

Analyses were performed in M*plus* 8.0. In a multilevel model, we first estimated standard random‐intercept models to test the simple effects of victimization and entity theory on depressive symptoms (H1), and a continuous interaction effect of Individual Victimization × Entity Theory (H2). Second, we tested the role of school‐average victimization in those patterns, using standard random‐intercept and random‐slope mixed‐effects models (Entity Theory × Individual × School‐average Victimization; H3). We did the latter by first estimating a model in which the random slopes were associations of an entity theory and peer victimization with depressive symptoms, which varied randomly across school‐level victimization (Individual Victimization × School‐average Victimization; Entity Theory × School‐average Victimization). Next, we also included the three‐way interaction with Individual Victimization × Entity Theory × School‐average Victimization. The equation for a final model including all two‐ and three‐way interactions of interest is presented below.

Level 1 (Student level):

$$\begin{array}{c} Y_{\mathrm{ij}}=\beta_{0j}+\beta_{1j}\left(Entity\;theory_{\mathrm{ij}}\right)+\beta_{2j}\left(Indiv\;Victimization_{\mathrm{ij}}\right)+\beta_{3j}\left(Entity\;theory_{\mathrm{ij}}\times Indiv\;Victimization_{\mathrm{ij}}\right)\\ +\beta_{4j}\left(Gender_{\mathrm{ij}}\right)+e_{\mathrm{ij}} \end{array}$$

Level 2 (School level):

$$\begin{array}{c} \beta_{0j}=\gamma_{00}+\gamma_{01}\left(Schoolwide\;Victimization_{j}\right)+\gamma_{02}\left(School\;Achievement_{j}\right)+\tau_{0j}\\ \beta_{1j}=\gamma_{10}+\gamma_{11}\left(Schoolwide\;Victimization_{j}\right)+\tau_{1j}\\ \beta_{2j}=\gamma_{20}+\gamma_{21}\left(Schoolwide\;Victimization_{j}\right)+\tau_{2j}\\ \beta_{3j}=\gamma_{30}+\gamma_{31}\left(Schoolwide\;Victimization_{j}\right)+\tau_{3j}\\ \beta_{4j}=\gamma_{40} \end{array}$$

When the significant interaction effect included school‐average victimization, we conducted follow‐up simple slope analyses. We did this by looking separately at “lower” victimization schools, which were schools where adolescents were on average rarely victimized (below the median of 1.33 out of 5‐point scale, *N = *2,999; *k* = 12) versus “higher” victimization schools, where adolescents were victimized on average at least a few times (equal to or above the median, *N = *3,238; *k* = 13). In both subsamples, we analyzed (1) how continuous associations between victimization and depressive symptoms differed across levels of the continuous measure of entity theory of personality, and (2) for visualization purposes the simple effects of victimization on depressive symptoms across incremental (<2 on entity theory scale: *N* = 1,108) versus entity (>4 on entity theory scale: N = 878) groups (Claro, Paunesku, & Dweck, 2016). We also included the covariates (gender, school achievement) in these analyses. The latter approach excluded data from a large number of participants (*N* = 4,251) who showed more average implicit theories. Therefore, as a sensitivity analysis, we replicated the results using a less strict, mean‐split approach for the implicit theories scale (below mean: *N* = 3,238; above mean: *N* = 2,999).

We controlled for gender and school achievement because we expected mean‐level differences in depressive symptoms between boys and girls and between schools that differ in academic achievement. Adolescent girls are often at risk for greater depressive symptoms than boys because they develop more social–emotional risk factors for depression than boys (Hankin & Abramson, 2001; Nolen‐Hoeksema & Girgus, 1994). Further, lower‐achieving schools may also have more students with greater levels of depressive symptoms (Huang, 2015). Despite these expected main effects on depressive symptoms, we did not expect that gender or school achievement would affect associations between implicit theories, victimization and depressive symptoms, or the role of context. In additional sensitivity analyses reported in Appendix 2, we controlled for potentially confounding effects of adolescents’ individual longer‐term history of victimization that mainly took place in their previous middle school (past *year* in eighth grade). The reason is that we aimed to examine how adolescents shape the meaning of individual victimization experienced in a current school context, and not their past experiences of victimization in a different context. In addition, in these sensitivity analyses, we examined potentially confounding effects of a measure of school quality that was used as an indicator of school safety in our validity analyses reported in Appendix 1.

Missing data were handled using full information maximum likelihood (FIML) estimation (Enders, 2010). Models using the total sample did not differ from those for only the subsample of adolescents with complete data. In all analyses, we used group‐mean centering of individual predictors (Level 1) and grand‐mean centering of the school‐average (Level 2) predictors, so that individual versus school‐average effects can be orthogonally estimated and residual errors are not confounded (Raudenbush & Bryk, 2002). Maximum likelihood estimation with robust standard errors (MLR) was used.

## Results

### Descriptive Statistics

Table 1 displays descriptive statistics and bivariate correlations for the central constructs of interest at the individual level; Table 2 includes these at the school level. On average, adolescents in the sample experienced low levels of depressive symptoms (never to sometimes), held a neutral entity theory of personality, thus between incremental and entity beliefs, and had been victimized only a few times in the past two weeks. However, there was variability in all three of these measures, and that variability was correlated, both on the individual level and the school level. On the individual level (Table 1), adolescents with higher levels of victimization (*r* = .42, *p* < .001) and higher entity theory of personality (*r* = .29, *p* < .001) also reported greater depressive symptoms. Those with higher levels of victimization also held more entity theory of personality (*r* = .23, *p* < .001).

**Table 1:** Individual‐Level Correlations among Study Variables

Variable	Correlations				Mean (*SD*)	Min–max
	1.	2.	3.	4.		
1. Depressive symptoms	–				0.41 (0.32)	0–2
2. Entity theory	.29***	–			2.94 (0.41)	1–6
3. Peer victimization	.42***	.23***	–		1.33 (0.55)	1–5
4. Gender	.19***	‐.01	.03	–	49% boys	0–1

Note

*N* = 6,237. Gender was dummy‐coded with female = 1, male = 0.

** *p* < .01;

*** *p* < .001.

**Table 2:** School‐Level Correlations among Study Variables

Variable	Correlations					Mean (*SD*)	Min–max
	1.	2.	3.	4.	5.		
1. Depressive symptoms	–					0.42 (0.01)	0.32 to 0.56
2. Entity theory	.45*	–				2.96 (0.03)	2.70 to 3.40
3. Peer victimization	.67***	.52**	–			1.34 (0.01)	1.22 to 1.47
4. School achievement	−.36	−.46*	−.16	–		1.63 (1.64)	−1.69 to 2.00
5. Gender	−.09	.38	.08	−.04	–	49% boys	0.31 to 0.61

Note

*N = *25*.*

* *p* < .05;

** *p* < .01;

*** *p* < .001.

On the school level (Table 2), schools in which students had on average higher depressive symptoms also had students with on average higher entity theory (*r* = .45, *p* = .024), higher victimization (*r* = .67, *p* < .001), and worse school prior achievement (*r* = −.36, *p* = .008).

### Multilevel Models

Estimating two‐level models, we examined the interplay between individual‐level victimization and entity theory on depressive symptoms, and how the average levels of victimization in adolescents’ school interacted with this pattern. Consistent with our preregistered planned analyses, we estimated consecutively the four standard random‐intercept models that each added one hypothesized effect: Model 1 only included the main effects on Level 1 of individual victimization and entity theory, Model 2 also included the Level 1 interaction between individual victimization and entity theory, Model 3 added the cross‐level interactions between school‐average and individual victimization and between school‐average victimization and entity theory, and the final Model 4 also included the three‐way cross‐level interaction between school‐average victimization (Level 2) × individual‐level victimization (Level 1) × entity theory (Level 1) on depressive symptoms. Since Model 4 included a significant 3‐way interaction, we interpret this model as the final model and will derive conclusions about the hypotheses based on this model (Table 3).

**Table 3:** Results of Multilevel Models Estimating Effects of Entity Theory, and Individual and School‐Average Victimization on Depressive Symptoms

	Final Model
	*b* (95% CI)
Level 1 (individual) (*N* = 6,237)	
Entity theory	0.07*** (0.06; 0.08)
Victimization	0.21*** (0.20; 0.23)
Gender	0.12*** (0.11; 0.13)
Entity Theory × Victimization	‐0.01 (−0.03; 0.02)
Level 2 (school) (k = 25)	
School‐average victimization	0.47*** (0.22; 0.70)
School achievement	0.00 (−0.00;0.00)
Cross‐level interactions	
Entity Theory × School‐average victimization	−0.02 (−0.18; 0.14)
Victimization × School‐average victimization	0.07 (−0.24; 0.38)
Entity Theory × School‐average victimization × Victimization	**0.29** * (**0.04; 0.50**)
Reduction compared to null model	
Reduction Level 1 residual variance	0.021 (22% reduction^a^)
Reduction Level 2 intercept variance	0.002 (33% reduction^b^)
Level 2 variability	
Entity theory	0.001
Victimization	0.000
Entity Theory × Victimization	0.001*

Note

The bold value underscores the three‐way interaction that tests the main hypothesis.

Unstandardized effects are shown. We controlled for clustering at the school level, and covariates were gender and school‐average achievement.

^a^ Null model residual variance = 0.095; final model residual variance* = *0.074.

^b^ Null model residual variance = 0.003; final model residual variance = 0.001.

* *p *< .05;

** *p* < .01;

*** *p* < .001.

As a preliminary matter, adolescents with higher levels of entity theory and victimization had higher levels of depressive symptoms (H1). However, the entity theory and individual‐level victimization interaction did not significantly predict levels of depressive symptoms overall (H2). The concurrent associations between individual students’ level of victimization and depressive symptoms were thus not moderated by adolescents’ implicit theories of personality if we did not take school context into account. Further, higher school‐level average victimization was associated with greater depressive symptoms, but school‐level victimization did not further exacerbate the extent to which individuals’ entity theory or victimization was associated with depressive symptoms.

Answering our primary question, and supporting H3, we found a significant School‐average Victimization (Level 2) × Individual‐level Victimization (Level 1) × Entity Theory (Level 1) cross‐level interaction effect on students’ depressive symptoms (*b* = 0.29, 95% *CI* [0.04–0.50], *p* = .013). These results were consistent with results from sensitivity analyses that included controlled for potentially confounding effects of adolescents’ individual longer‐term history of victimization that mainly took place in their previous middle school (past *year* in eighth grade) and an indicator of school quality (see Appendix 2).

To interpret this three‐way interaction, we conducted follow‐up analyses across schools that were lower in victimization (below the median) versus higher in victimization (above the median). First, tests of the Entity × Peer Victimization interaction across school contexts showed that in schools where victimization was more common (above the median), holding a higher entity theory exacerbated the extent to which higher peer victimization was related to greater depressive symptoms (*b = *0.02, *p* = .028, *R* ^2^ = *.*23). In schools with lower levels of victimization (below the median), by contrast, an entity theory did not moderate associations between peer victimization and depressive symptoms and the result was nonsignificantly in the opposite direction (*b =* −0.01, *p* = .147, *R* ^2^ = *.*21).

**Figure 1:**
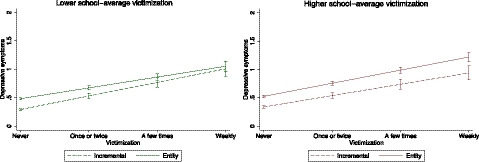
Predicted Results for Depressive Symptoms by Peer Victimization across Incremental (*n* = 1,108) and Entity Theory (*n* = 878), Lower (Average of Rarely Victimized) and Higher (Average of Victimized a Few Times) School‐Average Victimization. *Note.* Analyses are based on simple effects of peer victimization on depressive symptoms across incremental and entity theory groups in lower versus higher school‐average victimization schools.

**Figure A1:**
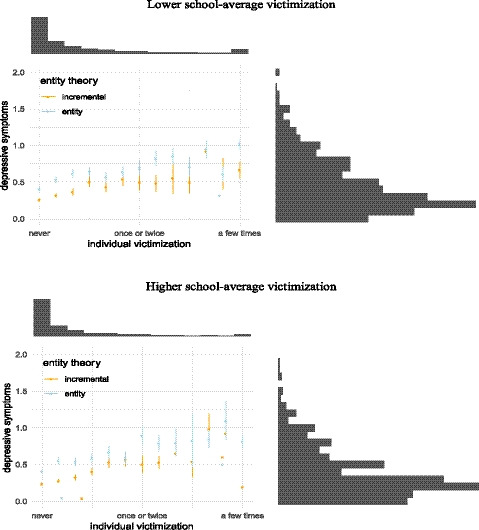
Graphic Representation of the Data Distribution in Both School Contexts (No Covariates Included). *Note*. We collapsed the answer categories of the victimization scale from “a few times” to “a few times per week” into one category because there were only a few observations in these highest categories.

The overall pattern of results for this three‐way interaction is depicted in Figure 1, which is based on estimates of simple effects of peer victimization on depressive symptoms across incremental and entity theory groups in lower (*b*_inc_ = 0.22, *b*_ent_ = 0.18) and higher (*b*_inc_ = 0.19, *b*_ent_ = 0.22) school victimization levels (the raw data are plotted in Figure A1). The patterns in Figure 1 show that, both in low‐ and high‐context victimization schools, individuals with an entity theory had highest levels of depressive symptoms. Finally, in another sensitivity analysis, patterns were in the same direction when using the less strict classification of incremental versus entity theory (the mean‐split approach of the entity theory scale; low = 3,238; high = 2,999), which uses data from all participants: for lower school‐average victimization, *b*_inc_ = 0.23, *b*_ent_ = 0.20, and for higher school‐average victimization, *b*_inc_ = 0.22, *b*_ent_ = 0.23.

## Discussion

The present study aimed to expand knowledge on individual social–cognitive processes that could be associated with depression, a known correlate of peer victimization, in interaction with school context. Previous experimental research has shown that adolescents’ beliefs about people’s potential for change (implicit theories) influence their emotional distress after encounters of exclusion and victimization (Rudolph, 2010; Yeager et al., 2014; Yeager, Trzesniewski, et al., 2013). More specifically, findings from those studies indicated that adolescents who held beliefs that people can change (*incremental* theory) experienced less distress and depressive symptoms after being victimized by peers, compared to adolescents who held beliefs that people’s socially relevant characteristics are nonmalleable (*entity* theory). However, we showed heterogeneity of these effects across social contexts, at least concurrently. Holding an entity theory of personality only exacerbated the associations between victimization and depressive symptoms in schools in which victimization was more common, thereby supporting the “*many fellow victims*” hypothesis. In schools where victimization was less common, adolescents who held an entity theory experienced greater depressive symptoms in general than those with an incremental theory but did not experience differential effects of victimization on depressive symptoms. Thus, as hypothesized, the relation between implicit theories and emotional correlates of victimization depends on the social context, namely how common victimization is in an adolescent’s school.

What explains the role of implicit theories in schools where adolescents have more fellow victims? We can only speculate. It is possible that socially adverse environments may create a social reality where victimization seems inevitable. This will be particularly threatening to adolescents with an entity theory, who believe that bad people like bullies cannot change. In high‐victimization contexts, the entity theory may be reinforced and supported by the world around you, which affords the fixed way of thinking (Walton & Yeager, 2020). Such high‐threat environments can relate to more hopelessness and depressive symptoms stemming from the perception that their victimization will persist.

The negative effect of hostile environments is reflected in earlier research on bullying and peer aggression, showing the negative mental health consequences of witnessing bullying at school for nonvictim students (Rivers et al., 2009) and becoming more aggressive from being in an aggressive environment (Henry et al., 2000). Being in a hostile environment can thus elevate feelings of threat for those who are individually victimized (Schacter & Juvonen, 2018b), especially for those who already have deterministic beliefs that they are unable to escape victimization. By contrast, adolescents who endorse an incremental theory, believing that it is possible for their victimization to change, may not consider having more fellow victims as a potent threat. This is in line with research showing that those with a more growth mindset have more adaptive coping strategies when they face adversity (Schroder et al., 2017). Moreover, it supports previous findings that implicit theories can have stronger impacts in contexts that afford those beliefs (Yeager et al., 2019).

Our findings also shed new light on recent studies that showed how victims might be protected against mental health problems when they were not alone (Brendgen et al., 2013; Garandeau et al., 2018; Huitsing et al., 2019; Schacter & Juvonen, 2018a). These studies on the “healthy context paradox” posit that having fellow victims could help victimized youth to emotionally cope with victimization (Huitsing et al., 2019; Salmivalli, 2018) and has been increasingly replicated by researchers. Being in a less safe environment characterized by more fellow victims can thus be more beneficial for victims’ emotional coping. The patterns observed in our study (at least in Figure 1) hinted at the possibility that having fellow victims was indeed associated with fewer depressive symptoms among victimized adolescents, but only among those who had more malleable beliefs and not among those with more nonmalleable beliefs about status‐relevant social traits. Thus, adolescents’ implicit beliefs are social–cognitive factors that could be considered when studying individual heterogeneity in the “healthy context paradox” phenomenon.

In schools where victimization was less common, implicit theories did not contribute to differential associations between victimization and depressive symptoms; those with an entity theory did not differ from those with an incremental theory in these associations. We did thus not find support for our “few fellow victims” hypothesis, which was based on the premise that ego threats exacerbate effects of implicit theories on emotional adjustment (Burnette et al., 2012) and that low‐victimization contexts can more strongly evoke ego threats because they strengthen self‐blaming, internal attribution processes (Schacter & Juvonen, 2016). We expected that adolescents who endorsed an entity theory perceived their experiences of failure as being fixed, and thus become more hopeless and depressed (Abramson & Metalsky, 1989), whereas those with an incremental theory would be less harmed by victimization in these contexts, because they may translate internal causal attributions to learning goals. Surprisingly however, our findings suggested a pattern in the opposite direction: that those who held an incremental theory in low‐victimization schools showed a stronger concurrent association between victimization and depressive symptoms compared to other groups, at least based on their steeper slopes. Although tentative given the nonsignificant effects of the continuous two‐way interaction, the pattern could suggest that attributing the cause of victimization to one’s own behaviors or characteristics still provides youth with an incremental theory, who are focused on improving their situation, with little information about what behaviors or traits they need to improve, and this lack of control may leave them more hopeless (Brown & Siegel, 1988; Sanjuán & Magallares, 2009). This aligns with previous evidence that those with an incremental theory need supportive contextual resources to be able to change their situation, and a low‐victimization context may provide victims with insufficient resources to do so (Yeager et al., 2019).

Overall, our findings are a first step in showing that adolescents may pay attention to the social context when they apply their implicit theories to make sense of their adverse social experiences. The central role of the peer context is particularly important in this developmental phase in which adolescents just made the transition to high school and try to understand the new social reality. Adolescents mainly look to their peers to interpret their own experiences (Brechwald & Prinstein, 2011; Killen, Rutland, Abrams, Mulvey, & Hitti, 2012), and when this peer context provides evidence of their implicit theories, it strengthens their impact (Yeager et al., 2019). As such, believing that people cannot change is associated with adverse mental health correlates when the peer context also signals that victimization is part of a common social reality that will likely perpetuate.

### Strengths and Limitations

To our knowledge, this study was the first to test the interactive role of school context and implicit theories of personality in predicting the links between peer victimization and concurrent depressive symptoms. In doing so, we relied on a U.S. national sample from 16 states that included information from more than 6,000 students in 25 public high schools. Moreover, we showed that our measure of school‐average victimization (aggregated with individual students’ self‐reports) was modestly associated with more objective indicators of peer aggression in schools. Despite the insights gained, our results need to be interpreted with some limitations in mind.

First, we could not test the temporal direction of the associations with our cross‐sectional design. It is therefore possible that depressive symptoms or implicit theories preceded victimization or that these are bidirectional processes. However, this does not affect our finding that the associations were only observed in schools where victimization was more prevalent and thus that the role of implicit theories is affected by context. Nevertheless, it is valuable to test whether effects of victimization on longitudinal changes in depressive symptoms over time can be affected by implicit theories, and whether such prospective associations are moderated by initial peer contexts in school.

Moreover, our victimization measure was limited to a time span of two weeks that also corresponded with the timescale of depressive symptoms. Although this allowed us to focus on more specific, short‐term effects, it is plausible that we underestimated the severity of victimization because it has been shown that being victimized monthly also contributes to maladjustment (Solberg & Olweus, 2003). Thus, future studies should replicate the analyses presented here to examine whether they are consistent when using different victimization measures, such as measures that use longer retrospective time spans in the same context. Regarding our school‐level measure of victimization, despite our validity analyses using objective criteria of school safety, it would still have been valuable to use a multi‐informant approach (e.g., including school‐based measures of victimization; or aggregates of parent‐ or teacher reports at school level) to address the problem of reference bias (Duckworth & Yeager, 2015).

### Suggestions for Future Research and Practical Implications

Our findings raised questions beyond the scope of the current research. Do the findings differ when focusing on aggression or revenge as behavioral outcomes? It has been shown that implicit theories can also influence associations between victimization and aggression or desires for vengeance following peer provocation (Yeager, Trzesniewski, et al., 2013). These patterns may also interact with context, for example, through greater hostile intent bias and greater vigilance to status threats (Lee & Yeager, 2019) among adolescents with an entity theory in contexts in which victimization and peer aggression are highly prevalent, and therefore, there exists greater needs to maintain vigilance to potential threats to their social status and regard.

Last, the findings also have implications for research on implicit theories. Although effects of implicit theories have been shown across multiple domains including intelligence, social and moral behaviors, internalizing and externalizing coping outcomes, and stress, health, and well‐being, to date the literature on implicit theories has predominantly focused on individual social–cognitive and coping processes (Yeager et al., 2019). However, according to our findings, effects of implicit theories on social adversity correlates can interact with the larger contexts in which adolescents are embedded. We hope this inspires researchers to consider other possible interactive effects between implicit theories and contextual factors as a way to understand how these psychological and sociological factors comold youth’s well‐being and mental health in the face of social adversity. More knowledge about the role of context is important to guide researchers in designing studies that are powered to detect effects (or not) within the subgroup of contexts that are expected to shape different construals of experiences (Tipton & Hedges, 2017; Walton & Yeager, 2020).

In addition to implications for future research, our study also has practical implications for implicit theories interventions, which should be interpreted with caution given the cross‐sectional nature of the study. Depending on the context, the meaning of “the potential for change” might be differentially contextualized. For instance, in schools with low victimization, an incremental theory of personality lesson might focus on reducing fixed, deficient self‐views (e.g., “I’m not a likable person”; “I will never be accepted”). In contrast, in high‐victimization contexts, incremental theories lessons might focus more on promoting the possibility of change for the whole context—for example, this school/ these peers can have a hope for change.

Overall, our findings implicate that adolescents’ mental health correlates of victimization are affected by the interplay between individual social–cognitive processes (implicit theories) and contextual factors that may give rise to different construals of socially adverse events. This raises awareness of the need for future longitudinal studies that can examine which mechanisms explain the different roles of implicit theories across school contexts. This is important to direct interventions aimed at reducing the harmful effects of peer victimization during adolescence.

## Appendix 1

### Analyses to Determine Validity of the Context‐Level Victimization Measure

- To determine the most valid operationalization of our school‐level victimization measure, we compared four possible ways of operationalization (the aggregated individual mean across all items; maximum score of all items—thus the highest score that a participant obtained across all items; and dichotomized mean/maximum score—thus the previous mean and maximum scores dichotomized using their mean) in their associations with publicly available data about the schools that could be considered indicators of social safety in each school (see our preregistration for details: https://osf.io/4qb5d). We focused exclusively on a measure of victimization in the last two weeks. Our preregistration also included a measure of victimization in the past year, but this time span seemed less suitable to understand effects of adolescents’ current context who attended a different school in the prior year.
- The four indicators of social safety in the school were (1) chronic absence, (2) suspensions, (3) school quality rating, the most recently available rating of school quality (all pulled from www.greatschools.org), and (4) incidents reported in ninth grade (collected among participants of the current study in the context of the *National Study of Learning Mindsets*).
- The mean score of victimization correlated strongest with the criteria, especially with more suspensions (*r* = .34, *p* = .10) and lower school quality rating (*r* = −.36, *p *= .08) (see Table A1).

**Table A1:** Validity Analyses of School‐Level Victimization: Pearson’s Correlations among Criteria and Past Two‐Week Victimization (6 items)

Variable	% Chronically Absent (*n = *24)	% Suspended (*n* = 25)	Great Schools (*n = *25)	Discipline Incidents (*n* = 12)
Mean	.04	.34^†^	−.36^†^	.14
Dichotomized mean	−.17	.16	−.20	.08
Maximum	−.01	.23	−.28	.14
Dichotomized maximum	−.17	.16	−.20	.08

^†^ *p < .*10.

## Appendix 2

### Consistent Findings with Victimization in Previous School as Covariate and Great Schools Rating as Covariate

We examined whether the results of the hypothesis tests were affected by victimization experiences in the previous school (i.e., past‐year victimization) or by school quality rating as potential confounders. Past‐year individual victimization was a maximum score of seven questions about victimization experiences during the last year (1 = *never* to 5 = *very often*): “I have been made fun of or disrespected because of my: (1) weight, (2) appearance, (3) school performance, (4) race, (5) family income, (6) religion, (7) sexuality or being gay/lesbian” (α = .81). A random 50% of participants answered these questions because they were exploratory. School quality rating was the variable used in the validity analyses (see Appendix 1). Results with the covariates included were similar for all hypotheses, compared to those that did not include these covariates (see Table A2).

**Table A2:** Results of Multilevel Models Estimating Effects of Entity Theory, and Individual and School‐Average Victimization on Depressive Symptoms Controlling for Victimization in the Previous School and School Quality Rating

	Final Model
	*b* (95% CI)
Level 1 (Individual) (*N* = 6,237)	
Entity theory	0.07*** (0.06; 0.08)
Victimization	0.19*** (0.17; 0.21)
Gender	0.12*** (0.10; 0.13)
Previous school victimization	0.06*** (0.04; 0.07)
Entity Theory × Victimization	0.00 (−0.02; 0.02)
Level 2 (School) (k = 25)	
School‐average victimization	0.46*** (0.22; 0.71)
School achievement	0.00 (0.00; 0.00)
School quality	0.00 (0.00; 0.01
Cross‐level	
Entity Theory × School‐average victimization	−0.01 (−0.12; 0.07)
Victimization × School‐average victimization	0.07 (−0.13; 0.28)
Entity Theory × School‐average victimization × Victimization	**0.26** * (**0.05; 0.47**)
Reduction in variance compared to null model	
Reduction Level 1 residual variance	0.011^a^
Reduction Level 2 intercept variance	0.001^b^
Level 2 variability	
Entity theory	0.001
Victimization	0.000
Entity Theory × Victimization	0.001*

Note

The bold value underscores the three‐way interaction that tests the main hypothesis.

Unstandardized effects are shown. We controlled for clustering at the school level, and covariates were gender, previous school victimization, and school‐average achievement.

^a^ Null model residual variance = 0.079; final model residual variance* = *0.068.

^b^ Null model residual variance = 0.002; final model residual variance = 0.001.

* *p* < .05;

** *p* < .01;

*** *p* < .001.

**Table A3:** Race/Ethnicity Demographics Within and Across Schools

School ID	Black/African American	Hispanic/Latinx	Native American Indian	White/European American	Asian/Asian American	Middle Eastern	Hawaiian/Pacific Islander
#	%	%	%	%	%	%	%
1	0.0	13.7	28.2	38.2	6.1	2.3	2.3
2	2.2	30.8	1.1	36.1	12.2	4.4	5.0
3	1.9	50.5	0.9	24.1	6.8	1.5	4.0
4	1.3	2.0	1.3	78.8	1.3	6.0	0.7
5	37.7	1.5	3.3	32.0	1.8	9.5	1.5
6	3.2	13.7	0.7	59.2	9.1	7.5	0.9
7	17.6	13.1	0.9	40.3	11.3	5.4	1.4
8	20.8	12.5	2.5	45.0	5.0	5.8	0.8
9	12.7	16.3	1.8	48.8	3.0	7.2	0.6
10	25.5	12.3	6.0	22.9	12.3	8.0	1.1
11	5.7	9.3	1.4	51.4	6.4	10.0	2.1
12	0.7	0.7	2.1	81.0	1.4	9.2	0.7
13	1.4	13.8	3.6	66.7	0.7	5.1	1.4
14	23.6	14.6	4.5	36.5	1.7	3.9	0.6
15	0.8	4.0	1.6	77.4	2.4	8.9	0.8
16	56.1	13.4	1.2	11.0	4.9	3.7	1.2
17	48.8	7.4	8.0	18.5	4.3	5.6	1.2
18	0.0	14.1	0.7	63.7	4.4	3.0	2.2
19	0.0	16.6	3.6	63.9	5.3	3.6	0.6
20	4.8	2.3	0.5	74.9	4.8	7.8	0.9
21	0.0	30.7	5.0	38.6	5.9	5.0	1.0
22	5.6	15.8	6.1	47.4	9.2	3.6	4.6
23	18.1	4.1	0.8	61.5	5.4	6.6	0.3
24	6.0	11.8	3.5	50.4	7.8	6.5	4.5
25	3.4	6.1	0.7	69.4	7.5	5.4	1.0
Mean	11.9	13.2	3.6	49.5	5.6	5.8	1.7

**Table A4:** Adolescents’ SES (Adolescent‐reported Highest Education of Mother) Within and Across Schools

School ID	No School	High School	Courses No College	AA/AS	BACHELOR	Master	PhD	Don't Know
	%	%	%	%	%	%	%	%
1	14.4	22.0	9.1	12.1	10.6	4.5	2.3	25.0
2	14.3	11.7	5.0	2.4	21.3	16.3	9.8	19.3
3	19.3	11.8	9.3	2.5	14.0	10.9	5.6	26.5
4	0.7	11.8	9.9	3.9	23.0	25.0	4.6	21.1
5	16.8	21.2	11.5	6.9	10.9	5.3	1.6	25.9
6	8.7	14.4	8.4	4.8	25.8	16.7	2.7	18.5
7	16.9	20.1	13.2	5.5	9.1	4.6	2.3	28.3
8	13.6	11.9	12.7	6.8	18.6	8.5	2.5	25.4
9	8.5	11.6	14.6	6.7	26.2	6.7	1.2	24.4
10	10.0	17.1	10.9	7.7	19.4	8.9	3.7	22.3
11	16.7	9.4	13.8	6.5	15.9	6.5	2.9	28.3
12	1.4	16.8	10.5	7.7	27.3	13.3	4.2	18.9
13	8.8	11.8	10.3	8.1	22.1	11.8	2.9	24.3
14	16.6	13.7	12.6	12.0	9.7	5.7	4.0	25.7
15	7.3	22.6	11.3	11.3	16.9	5.6	3.2	21.8
16	20.5	21.8	7.7	3.8	6.4	5.1	1.3	33.3
17	12.6	20.8	13.8	13.8	9.4	2.5	4.4	22.6
18	11.3	15.8	12.0	12.0	9.0	7.5	2.3	30.1
19	9.1	18.2	12.1	9.7	16.4	8.5	0.6	25.5
20	2.8	13.6	9.7	6.9	27.1	18.4	3.7	17.9
21	7.9	15.8	17.8	5.0	21.8	9.9	2.0	19.8
22	5.2	18.2	14.1	9.9	15.6	6.3	1.6	29.2
23	3.6	11.4	8.8	7.2	25.8	15.2	4.4	23.5
24	5.6	16.0	11.2	7.1	19.3	10.2	2.3	28.4
25	5.1	12.7	8.2	7.9	29.8	15.1	6.2	15.1
Mean	10.3	15.7	11.1	7.5	18.1	10.0	3.3	24.0
